# Cell-based analysis of Chikungunya virus E1 protein in membrane fusion

**DOI:** 10.1186/1423-0127-19-44

**Published:** 2012-04-21

**Authors:** Szu-Cheng Kuo, Ying-Ju Chen, Yu-Ming Wang, Pei-Yi Tsui, Ming-Der Kuo, Tzong-Yuan Wu, Szecheng J Lo

**Affiliations:** 1 Division of Microbiology, Graduate Institute of Biomedical Sciences, College of Medicine, Chang Gung University, TaoYuan, Taiwan; 2 Institute of Prevention Medicine, National Defense Medical Center, Taipei, Taiwan; 3 Department of Bioscience Technology, Chung Yuan Christian University, Chung-Li, Taiwan

**Keywords:** Alphavirus, Bicistronic baculovirus expression system, Chikungunya virus, Class II fusion protein, Fusion peptide, Membrane fusion

## Abstract

**Background:**

Chikungunya fever is a pandemic disease caused by the mosquito-borne Chikungunya virus (CHIKV). E1 glycoprotein mediation of viral membrane fusion during CHIKV infection is a crucial step in the release of viral genome into the host cytoplasm for replication. How the E1 structure determines membrane fusion and whether other CHIKV structural proteins participate in E1 fusion activity remain largely unexplored.

**Methods:**

A bicistronic baculovirus expression system to produce recombinant baculoviruses for cell-based assay was used. Sf21 insect cells infected by recombinant baculoviruses bearing wild type or single-amino-acid substitution of CHIKV E1 and EGFP (enhanced green fluorescence protein) were employed to investigate the roles of four E1 amino acid residues (G91, V178, A226, and H230) in membrane fusion activity.

**Results:**

Western blot analysis revealed that the E1 expression level and surface features in wild type and mutant substituted cells were similar. However, cell fusion assay found that those cells infected by CHIKV E1-H230A mutant baculovirus showed little fusion activity, and those bearing CHIKV E1-G91D mutant completely lost the ability to induce cell-cell fusion. Cells infected by recombinant baculoviruses of CHIKV E1-A226V and E1-V178A mutants exhibited the same membrane fusion capability as wild type. Although the E1 expression level of cells bearing monomeric-E1-based constructs (expressing E1 only) was greater than that of cells bearing 26S-based constructs (expressing all structural proteins), the sizes of syncytial cells induced by infection of baculoviruses containing 26S-based constructs were larger than those from infections having monomeric-E1 constructs, suggesting that other viral structure proteins participate or regulate E1 fusion activity. Furthermore, membrane fusion in cells infected by baculovirus bearing the A226V mutation constructs exhibited increased cholesterol-dependences and lower pH thresholds. Cells bearing the V178A mutation exhibited a slight decrease in cholesterol-dependence and a higher-pH threshold for fusion.

**Conclusions:**

Cells expressing amino acid substitutions of conserved protein E1 residues of E1-G91 and E1-H230 lost most of the CHIKV E1-mediated membrane fusion activity. Cells expressing mutations of less-conserved amino acids, E1-V178A and E1-A226V, retained membrane fusion activity to levels similar to those expressing wild type E1, but their fusion properties of pH threshold and cholesterol dependence were slightly altered.

## Background

Chikungunya virus (CHIKV) is an enveloped plus-stranded RNA virus classified into the genus Alphavirus of family Togaviridae. The CHIKV genomic organization is arranged as with other alphaviruses: "5'-nsP1-nsP2-nsP3-nsP4-junction region-C-E3-E2-6K-E1-poly (A)-'3 " [1]. The first open reading frame (ORF) of the genome encodes a polyprotein to yield all non-structural proteins nsP1 to nsP4. A subgenomic RNA containing the second ORF encodes a polyprotein that produces the structural proteins, including capsid protein (C) and two envelope proteins (E2 and E1) [1]. E2 and E1 are glycoproteins embedded in the viral membrane in a heterodimeric form and are responsible for viral attachment and membrane fusion, respectively [2]. Viral membrane fusion with a cell membrane is mediated by the E1 glycoprotein, a class II fusion protein [3-5], in a process dependent on low-pH. Acidic conditions induce a conformational change in the viral envelope proteins, dissociation of the E2-E1 heterodimers, and formation of E1 homotrimers [6-8]. The E1 trimer inserts into the target membrane via its hydrophobic fusion peptide and refolds to form a hairpin-like structure [9,10]. In addition to the dependence on low pH for viral membrane fusion, cholesterol is also required for both cell membrane fusion and budding during alphavirus infection [11-14].

Chikungunya fever manifests after a bite by a CHIKV-infected *Aedes* mosquito, and is associated with clinical symptoms including fever, headache, myalgia, and joint pains [15]. An expanding worldwide pattern of CHIKV epidemics has been reported [16]. A recent E1-A226V mutant virus outbreak emerged in the Indian Ocean where the virus had adapted to a broadly distributed vector, *A. albopictus*[17-19]. The CHIKV viral infection's geographical expansion was facilitated by E1-A226V mutant expressing high vector competence towards *A. albopictus*. E2-I211T and E1-T98A mutants have an epistatic interaction with the E1-A226V mutation that influences CHIKV fitness in *A. albopictus *[20,21]. Additional lines of evidence from related alphavirus, Semliki forest virus (SFV) and Sindbis virus (SINV), show that the valine residue at position 226 of the E1 glycoprotein, controls the cholesterol dependence of viral-cell membrane fusion [22,23]. Thus, the CHIKV E1-A226V mutation has the potential to alter E1's biological properties including fusogenicity, which may explain the adaptation mechanism seen in *A. albopictus*.

Viral fusion proteins contain a "fusion" amino acid sequence. For example, a recent study demonstrated that the HIV fusion peptide at the gp41 N-terminus, a glycine-rich hydrophobic sequence, is a critical determinant of HIV-infected cell membrane fusion [24]. Substitution of glutamic acid for glycine in the fusion sequence of influenza virus HA2, prevents cell fusion in simian cells [25]. In alphaviruses, E1 also contains a highly conserved fusion peptide within a hydrophobic region of 19 amino acids that span amino acid residues 83 through 101 [26]. SFV E1-G91D, a glycine within the fusion peptide loses its fusion activity on substitution with glutamic acid [27]. However, SFV E1 *srf-3* (sterol requirement in function) mutant (P226S) and *srf-5 *mutant (V178A), two substituted amino acids located outside of fusion sequences, were identified by cholesterol-depletion experiments in mosquito cell lines; both mutants show decreased fusogenicity related cholesterol-dependence [22,28]. The recently identified CHIKV A226V mutant may be selected by the adaptation to *A. albobilicus*[17,29,30]. Although the crystal structures of CHIKV glycoproteins have been determined, the detailed functional relationship between structure and fusogenicity on CHIKV E1 protein remains unclear [26].

To explore the structure and fusogenicity of the E1 protein, four E1 residues (G91, V178, A226, and H230) were selected for change by mutation. Mutants were tested for their ability to induce cell membrane fusion. Constructs expressing the wild type protein E1, or a single-amino-acid substitution, either in monomeric-E1 form or with two other structural proteins (capsid protein C and envelope protein E2), were introduced into a bicistronic baculovirus expression system [32] to produce recombinant baculoviruses for cell-based assay [31]. A bicistronic baculovirus expression system co-expressing CHIKV structural proteins and EGFP (enhanced green fluorescence protein) easily identified EGFP-positive Sf21 cells that simultaneously expressed E1 and E2 on the cell surfaces [31]. Use of this system facilitated our analysis of the E1 fusogenicity determinant, and revealed that changes in E1 conserved amino acids (G91 and H230) resulted in losing all fusogenicity.

## Methods

### Site-directed mutagenesis and plasmid constructs

**Figure 1:**
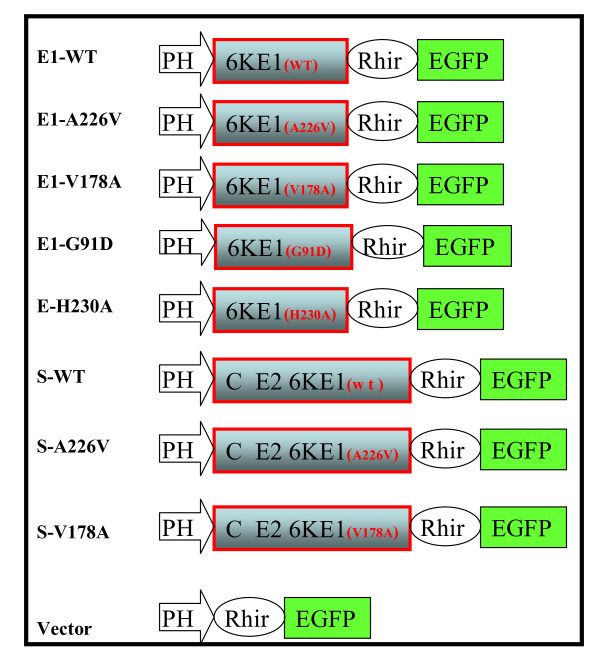
**Schematic representation of plasmids for production of recombinant baculoviruses bearing a variety of CHIKV E1 mutants**. Expression of CHIKV 6K-E1 protein (monomeric-E1 based constructs): E1-WT, E1-A226V, E1-V178A, E1-G91D and E1-H230A contain a 6K-E1 region cDNA fragment and the indicated site for substitutions (line 1 to line 5, respectively). For expression of all of the CHIKV structural polyproteins (26S-based constructs): S-WT, S-A226V, and S-V178A contain the full-length cDNA of the CHIKV 26S subgenomic RNA (AF369024) and indicated mutations (line 6 to line 8). Abbreviations: PH = polyhedrin promoter; CE26KE1 = a polyprotein translated from CHIKV 26S RNA; EGFP = enhanced green fluorescent protein gene; Rhir = RhPV 5'-UTR IRES.

DNA preparations and manipulations were performed as described by Sambrook et al. [33] or following protocols provided by reagent manufacturers. The backbones of baculovirus transfer vectors, pBac-CHIKV-26S-Rhir-E and pBac-CHIKV-6K-E1-Rhir-E containing the full-length cDNA of CHIKV 26S subgenomic DNA and 6K-E1 region respectively, were previously characterized [31]. For simplicity, we named the former plasmid the "26S-based construct" (pS-WT, see Figure 1), and the latter the "monomeric-E1-based construct" (pE1-WT, see Figure 1). Mutagenesis was performed (Quick Change site-directed mutagenesis kit, Stratagene, La Jolla, CA) to create single amino acid substitutions (pE1-A226V, pE1-V178A, pE1-G91D, and pE1-H230A). The 973-bp NheI-PstI fragment of pS-WT was substituted by a 973-bp NheI-PstI fragment containing a V178A mutation to provide pS-V178A, and substituted by a 2-kb NcoI fragment to provide pS-A226V. All constructs were confirmed by DNA sequencing.

### Recombinant virus production and titer determination

The production and titer determination of recombinant viruses were as described previously [31]. Baculoviruses derived from monomeric-E1-based constructs were named vE1-WT, vE1- A226V, vE1-V178A, vE1-H230A, and vE1-G91D, while those derived from 26S-based constructs were named vS-WT, vS-A226V, and vS-V178A. The recombinant viruses were purified by a series of three end-point dilutions. Sequences of all recombinant viruses were confirmed by viral DNA sequencing. In brief, viral genomic DNAs of recombinant baculoviruses were purified using a QIAamp genomic DNA purification kit (Qiagen, Hilden, Germany), and then used as templates for PCR amplification using Phusion High-Fidelity DNA Polymerase. The amplified DNA fragments were sequenced using the dideoxy chain-termination method.

### Cell surface biotinylation

Cell surface proteins were biotinylated using literature methods [34]. Sf-21 cells, an insect cell line commonly used for baculovirus infection, were seeded at 2 × 10^6 ^cells/well in a 6-well plate and infected with recombinant baculoviruses at a multiplicity of infection (M.O.I.) of one in Sf-900 II SFM, containing 8% fetal calf serum (FCS) at pH 6.4. At two days post infection (dpi), cells were washed twice with PBS (2 ml, pH 7.4), then incubated with 200 μl PBS (pH 7.4) containing EZ-Link Sulfo-NHS-LC-Biotin (0.5 mg/ml) (Pierce Chemical, Rockford, IL) at 4°C for 30 minutes. The incubated cells were washed twice with glycine in PBS (2 ml, 100 mM, pH 7.1) to stop biotinylation. Cells were then lysed in 250 μl RIPA buffer (Thermo Scientific, Rockford, IL) containing an EDTA-free Protease Inhibitor Cocktail (Roche, Mannheim, Germany). The lysates were sonicated on ice using a Microson XL 2005 ultrasonic cell disruptor equipped with a P1 microprobe (Heat Systems Inc., NY) at 14 W for 10 seconds. Samples were then centrifuged at 15000 rpm for 15 minutes. The resulting pellets were dissolved into urea (200 μl, 8 M) and re-centrifuged at 15000 rpm for 15 minutes to remove un-dissolved materials. Fifty-microliter aliquots of the supernatant were mixed with PBS (350 μl, pH 7.4). Biotinylated cell surface proteins in supernatant were separated from non-biotinylated proteins by precipitation with 50 μl M-280 streptavidin magnetic beads (Invitrogen Dynal AS, Oslo, Norway) at 4°C for 60 minutes. Beads were washed three times with 0.5 ml PBS. Biotinylated E1 proteins were then eluted by boiling the beads in 50 μl 2 × Laemmli sample buffer containing 4 M urea, resolved by 10% SDS-PAGE, and detected by Western blot using rabbit anti-CHIKV E1. The same blot was re-probed by mAb anti-gp64 (AcV5 sc-65499; Santa Cruz, CA) as a control for total protein loading.

### Western blot analysis

Western blot analysis was performed as previously reported [31] with modifications. Sf21 cells were seeded at 1 × 10^6 ^cells/well in a 24-well plate and infected with recombinant viruses at an M.O.I. of one. Total proteins harvested at 2 dpi were dissolved in Laemmli sample buffer containing 4 M urea and separated onto 10% SDS-PAGE. The blotted membranes were incubated with rabbit anti-CHIKV E1 serum (1/500), anti-CHIKV E2 serum (1/500), or anti-gp64 monoclonal antibody (1/2000) (Santa Cruz Biotechnology, Inc, Santa Cruz, CA). The membranes were then incubated at a 1:1000 dilution with Peroxidase-conjugated Goat anti-Rabbit IgG (Jackson ImmunoResearch Laboratories, Inc., Philadelphia, Pa) or peroxidase-conjugated anti-mouse IgG (KPL, Gaithersburg, MD) for one hour at room temperature. Peroxidase was detected on the membrane using a LumiFast Plus Chemiluminescence Detection Kit (T-Pro Biotechnology, Taiwan, ROC) following the manufacturer's protocol. The UVP AutoChemi Image System was used for capturing and processing the various images.

### Immunofluorescence microscopy

For CHIKV E1 protein staining the cell surface, Sf21 cells were seeded in a 96-well plate and infected with recombinant baculoviruses at an M.O.I. of one in Sf-900 II (pH 6.4) containing 2% FCS. At two dpi, cells were fixed with 3% formaldehyde and stained with rabbit anti-whole CHIKV serum at a dilution of 1:100 for 30 minutes at room temperature. After washing twice with cold PBS, cells were incubated with the secondary antibody, Alexa Fluor 594-conjugated goat anti-rabbit IgG (Invitrogen, Molecular Probes, Carlsbad, CA) at a dilution of 1:500 for 30 minutes at room temperature and then washed twice with cold PBS. The stained cells were recorded using an inverted fluorescence microscope (Olympus Model IX71, Tokyo, Japan), red-channel for CHIKV E1 staining and green-channel for EGFP.

### Cell-cell fusion assay

CHIKV E1 fusion activity, expressed on the baculovirus-infected Sf21 cell surfaces was examined using a cell-cell fusion assay as previously described [31]. Briefly, Sf21 cells were infected with indicated recombinant baculoviruses at an M.O.I. of one in Sf-900 II SFM, with or without FCS. After one dpi, the culture medium was replaced with Sf-900 II SFM containing either 2% FCS, cholesterol (100 μg/ml) and indicated pH levels for pH-dependence assay, or containing indicated concentrations of cholesterol for cholesterol-dependence assay. The syncytial formations were examined and photographed using an inverted fluorescence microscope (Olympus Model IX71, Tokyo, Japan). Calculation of the fusion index was modified from previous reports [25,35]. Briefly, the average size of a single cell was determined by the formula: Total area of 100 Sf21 cells/100. The number and area of single-cell or syncytia were counted and measured using ImageJ software [36]. The number of EGFP positive nuclei was calculated by: total areas containing at least 100 cells/average single-cell size. The fusion index was calculated using: 1-(number of EGFP positive cells/number of EGFP positive nuclei). In the comparisons of syncytial cell size, cell number, and total area, at least 100 EGFP positive single cells were counted and measured. The average size of syncytial cells was calculated as: total area/number of cells.

## Results

### Construction of recombinant baculovirus vectors that express mutant E1 protein

**Figure 2:**
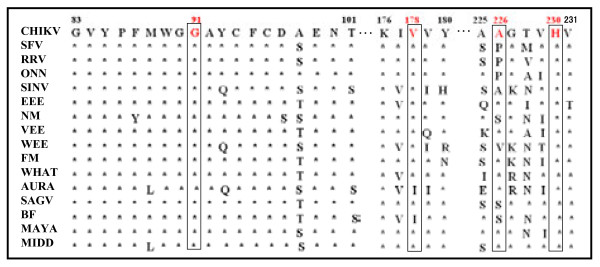
**Conservation of four key amino acids of CHIKV E1 protein among 16 alphaviruses**. Four amino acids (G91, V178, A226, and H230 shown in red and boxed) were investigated to characterize their role in E1 membrane fusion. Flanking amino acids are shown in three segments, segment 1: amino acids 83 to 101 containing the fusion peptide region, segment 2: amino acid 176 to 180, and segment 3: amino acid 225 to 231. Amino acids are shown with a single letter abbreviation, and the identical amino acid is shown as an asterisk. Abbreviations of viruses and GenBank accession numbers held by the National Center for Biotechnology Information Database are as follows: CHIKV, Chikungunya virus (AF369024); SFV, Semliki Forest virus (X04129); RRV, Ross River virus (M20162); ONN, O'Nyong-nyong virus (AF079456); SINV, Sindbis virus (P89913); EEE, Eastern equine encephalitis virus (Q9PZX1); NM, Ndumu virus (AAL35778); VEE, Venezuelan equine encephalitis virus (Q9YKC9); WEE, Western equine encephalitis virus (Q9IBP3); FM, Fort Morgan virus (Q80S49); WHAT, Whataroa virus (Q80S41); AURA, Aura virus (Q86925); SAGV, *Sagiyama *(AAO33337.1); BF, Barmah Forest virus (AAO33347), MAYA, Mayaro virus (AAO33335.1), and MIDD, Middleburg virus (AA033343.1).

The roles in membrane fusion activity and cholesterol requirement of four amino acid residues at positions 91, 178, 226, and 230 in SFV and SINV E1 proteins have been reported [22,23,27,37]. However, the roles of these amino acid residues in CHIKV E1-mediated viral membrane fusion have not yet been characterized. To determine the conservation of the four amino acid residues across known alphaviruses, a partial sequence of CHIKV E1 containing the four residues was aligned with those sequences in 15 other alphaviruses: Semliki Forest virus, Ross River virus, O'Nyong-nyong virus, Sindbis virus, Eastern equine encephalitis virus, Ndumu virus, Venezuelan equine encephalitis virus, Western equine encephalitis virus, Fort Morgan virus, Whataroa virus, Aura virus, Sagiyama, Barmah Forest virus, Mayaro virus, and Middleburg virus (Figure 2). G91, and H230 were conserved in all listed alphaviruses, and CHIKV E1 V178 was substituted by I178 (isoleucine-178) in Aura virus and Barmah Forest virus. However, CHIKV E1 A226 was less conserved, and could be represented by proline, serine, or valine in other viruses (Figure 2).

To evaluate the influence of residues at positions 91, 178, 226, and 230 of E1 protein on cell fusion, we used site-directed mutagenesis to generate pE1-A226V, pE1-V178A, pE1-G91D, and pE1-H230A mutants in the CHIKV E1 gene. Mutants were cloned into the bicistronic baculovirus expression vector: pBac-Rhir-E (Figure 1 bottom line), which only expresses E1 protein. E1-A226V and E1-V178A mutations were also introduced into 26S-based constructs, which express all structural proteins, to furnish pS-A226V and pS-V178A (Figure 1), respectively. These clones were used to investigate possible effects of other CHIKV structural proteins on E1-mediated membrane fusion.

### Synthesis and cell surface expression of wild type and mutant E1 protein

**Figure 3:**
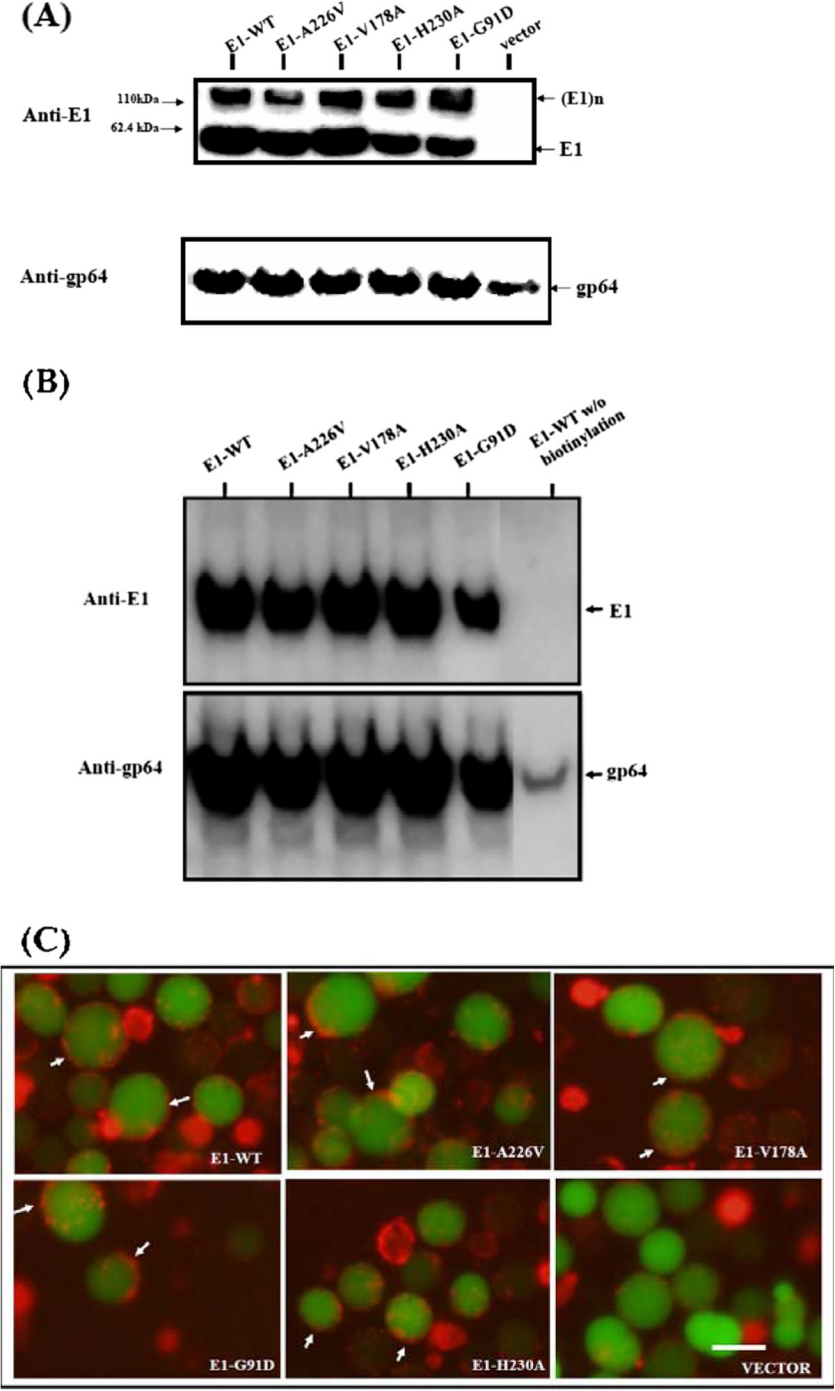
**Expression of CHIKV E1 proteins in Sf21 cells infected by recombinant baculoviruses**. **(A) **Sf21 insect cells were grown and infected with recombinant viruses at an M.O.I. of one. Total protein was harvested at 2 dpi and separated on 10% SDS-PAGE. Viral glycoproteins were detected by Western blot using rabbit anti-CHIKV E1 serum (upper gel), then re-probed with anti-baculovirus gp64 antibody (lower gel). Two protein size markers are indicated on the left. **(B) **Detection of E1 on the cell surface by biotinylation assay. Baculoviruses infected-Sf21 cells as indicated were labeled with biotin and lysed. Biotinylated surface proteins were resolved by 10% SDS-PAGE and detected by Western blot using rabbit anti-CHIKV E1 serum (upper blot), and then re-probed with anti-baculovirus gp64 antibody (lower blot). Cell conditions and protein markers are given by the legend of **(A)**. **(C) **Immunofluorescence analysis of CHIKV E1 on the cell surface. Sf21 cells infected with the indicated recombinant baculoviruses were stained with anti-CHIKV E1 antibodies followed by secondary goat anti-rabbit IgG antibodies labeled with Alexa Fluor 546. Cells were examined and photographed using a fluorescent microscope under identical green and red lighting conditions. Overlaid images show green fluorescence representing the infected-Sf21 cells expressing EGFP, and red fluorescence representing CHIKV E1 protein signals (indicated by arrows). Cells without EGFP, but stained in red were dead cells. The bar represents 10 μm.

Recombinant baculoviruses carrying wild type or substituted E1 gene were generated as described in Methods. An insect cell line, the Sf21 cell, was infected by recombinant baculoviruses. Infected Sf21 cells emitted green fluorescence, and cell lysates were subjected to Western blot analyses by anti-E1 polyclonal antibodies. Protein bands migrating to the 60 kDa and 120 kDa gel positions, corresponding to the monomeric and dimeric forms of E1, were detected in cells infected by baculoviruses containing wild-type or single-amino-acid substitution mutant of E1, but not the control vector (Figure 3A, upper blot). These results indicate baculovirus infected Sf21 cells can express the four mutant E1 proteins with single-amino-acid substitutions, in addition to expressing wild type E1 protein. Comparison with the gp64 loading control band (a baculovirus glycoprotein) showed that wild type and substituted form E1 proteins were expressed in similar quantities (Figure 3A, lower blot).

To test whether these E1 proteins were targeted to the cell surface, we performed biotin labeling on the cell surface followed by Western blot analysis. Results showed that both E1 proteins (wild type or substituted form) and the gp64 control protein were labeled with biotin (Figure 3B), indicating that all forms of E1 protein were located on the cell surface. To confirm these results, we conducted immunofluorescence microscopy using antibodies conjugated with Alexa Fluor 546 to detect the E1 cell surface. Figure 3C shows that red signals, from E1 present at the cell surface, either in wild type or in mutants, were observed for cells infected by the corresponding recombinant baculovirus under a fluorescence microscope (indicated by arrows). However, no red signal was detected in cells infected by control baculoviruses (lower right panel), indicating that both wild type and mutant E1 proteins were targeted to the cell surface.

### Fusion activities and properties of wild type and mutant E1 expressed by monomeric-based constructs

**Figure 4:**
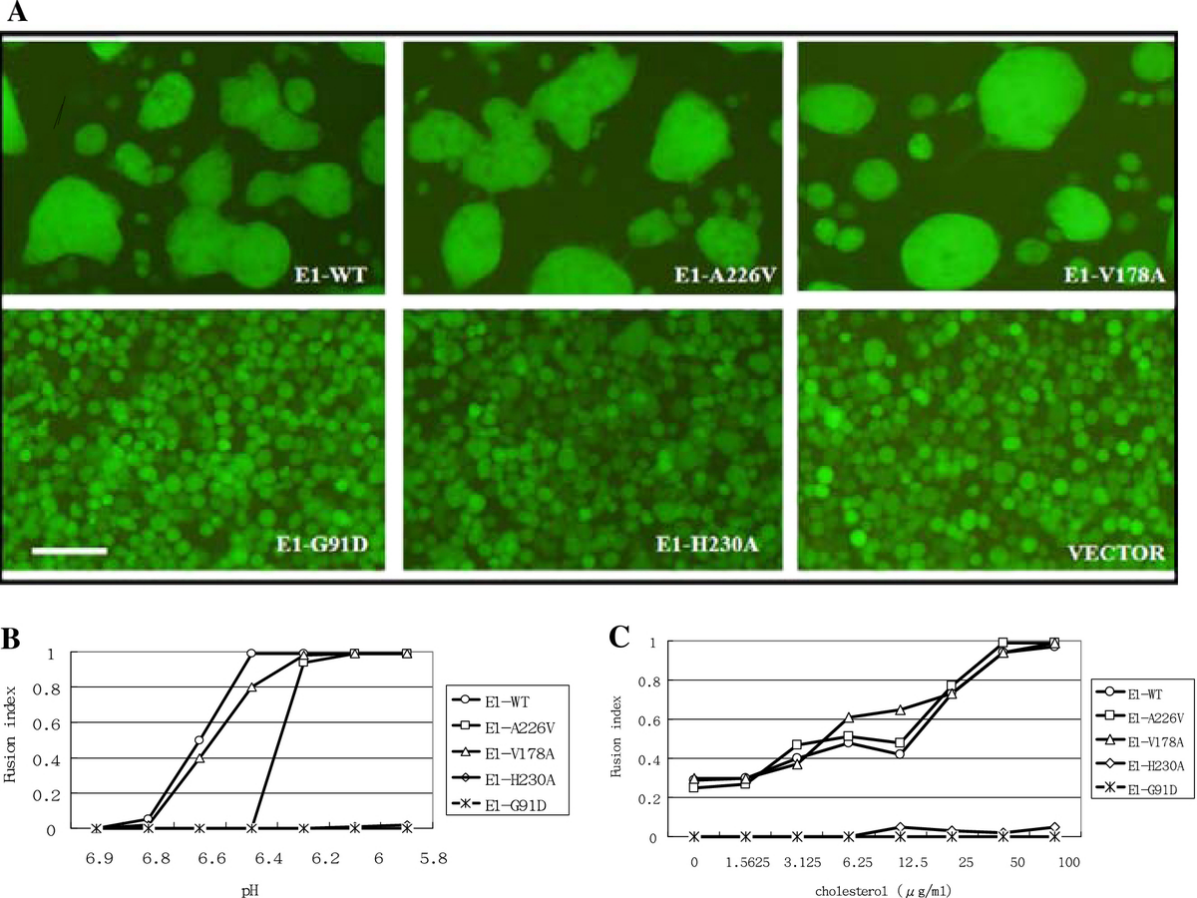
**pH and cholesterol dependency in cells induced by monomeric-E1-based constructs**. **(A) **Sf21 cells were infected with the indicated recombinant baculoviruses. After 1 dpi, the culture medium was replaced with Sf-900 II SFM (pH = 5.8) containing 2% FCS and 100 μg/ml cholesterol to triggering cell-cell fusion. Syncytial formation was examined under a fluorescence microscope with green channel. The bar represents 50 μm. **(B) **pH profiles of cell fusion induced by wild type or mutant E1. Sf21 cells were infected with the indicated recombinant baculoviruses. After 1 dpi, the culture medium was replaced with Sf-900 II SFM containing 2% FCS, 100 μg/ml cholesterol and pH levels of 5.8, 6.0, 6.2, 6.4, 6.6, 6.8, and 6.9 as indicated. Syncytial formation was examined under a fluorescence microscope. At least 100 nuclei per field were counted at a 200× magnification. The fusion index was determined using: Number of multiple nuclei cells/number of EGFP positive cells. **(C) **Cholesterol-dependent profiles. Sf21 cells that passaged at least three times in Sf-900 II SFM were infected with the indicated recombinant baculoviruses. After 1 dpi, the culture medium was replaced with Sf-900 II SFM (pH = 5.8) containing 2% FCS and, various levels of cholesterol as indicated. Syncytial formation was examined under a fluorescence microscope. Approximately 100 nuclei per field were counted at 200× magnification. The fusion index was determined as described above.

To examine whether single-amino-acid substitution mutants retain the ability to mediate membrane fusion, Sf21 cells, infected by various recombinant baculoviruses were incubated under optimal conditions for CHIKV E1-mediated membrane fusion, in a medium containing 100 μg/ml cholesterol at pH 5.8 [31]. Green fluorescence positive cells in syncytium were examined under a fluorescence microscope. No cell fusion was observed in those cells infected by control baculoviruses or baculoviruses bearing E1-G91D (Figure 4A, lower right and left panels). Only few, and small, fusion cells were found in cells expressing E1-H230A (Figure 4A, lower middle panel), indicating that substitution of the conserved amino acid in E1 lost the most membrane fusion ability compared to other cells. However, cells expressing E1-A226V and E1-V178A showed fusion similar to cells that express wild-type E1 protein (Figure 4A, top panels), indicating that substitution of less-conserved residues of E1 did not eliminate fusogenicity.

To analyze whether E1-A226 and E1-V178 protein's pH and cholesterol dependency differ from those of wild-type E1 protein, cells were incubated with medium containing 200 μg/ml cholesterol at various pH values (ranging from pH 5.8 to pH 6.9), or at a constant pH of 5.9 with varying concentrations of cholesterol (ranging from 0 to 100 μg/ml). Cell fusion capacity was analyzed by fluorescence microscopy and fusion indexes were determined. To attain 100% cell fusion, cells expressing E1-A226V required a slightly lower pH than those expressing wild type E1 or E1-V178A (pH = 6.2 vs. pH = 6.4) (Figure 4B). Cells expressing wild type E1, E1-V178A, and E1-A226V required 50 μg/ml cholesterol to achieve 100% cell fusion. Nevertheless, cholesterol dependence was lower in cells expressing E1-V178A to attain 60% cell fusion, compared to cells expressing either wild-type E1 or E1-A226V (Figure 4C). No cell fusion was observed in cells expressing G91D or H230A (Figure 4C).

## E1 co-expression with other CHIKV structural proteins exhibits different membrane fusion properties

**Figure 5:**
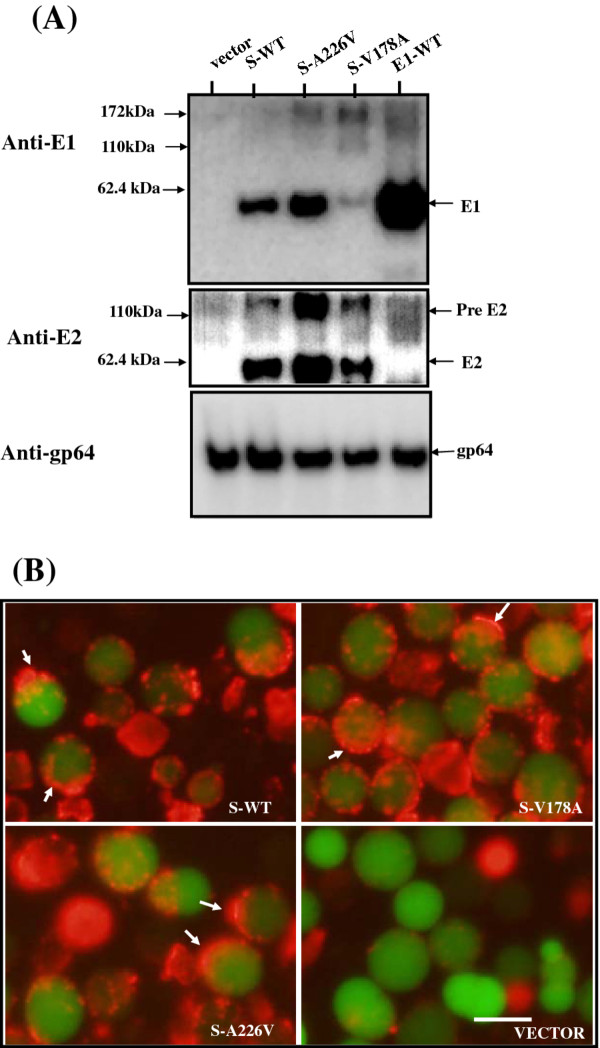
**E1 co-expression with other structural proteins by 26 S-based constructs**. Sf21 cells were grown in a six-well plate and infected with recombinant viruses at an M.O.I. of one. E1 and E2 proteins were detected by Western blot analysis using rabbit anti-CHIKV E1 serum (upper gel), or anti-CHIKV E2 serum (middle gel) then re-probing with anti-baculovirus gp64 antibody (lower gel). Proteins extracted from cells were infected with various baculoviruses as indicated above the gel. Arrows on the right indicate the CHIKV E1 and E2 proteins. Two protein size markers are shown on the left. (B) Immunofluorescence analysis of CHIKV E1 on the cell surface. Sf21 cells infected with the indicated recombinant baculoviruses and described as above were stained as per Figure 3C. The bar represents 10 μm.

E2 plays a role in E1 fusogenicity with other alphaviruses; we infected cells with baculovirus constructs expressing the C protein E2, and wild type or mutant E1, and examined the cell fusion capability. Cells infected by recombinant baculoviruses were first analyzed for expression of E2 and E1. Western blot results showed that E2 was expressed in cells infected by baculoviruses bearing S-WT, S-A226S, and S-V178A, but not in cells bearing vector or monomeric-E1 base (E1-WT) (Figure 5A, center of gel panel). However, for unknown reasons, the E1 expression level from cells infected by baculoviruses bearing S-V178A was much lower than it was for those cells bearing S-WT and S-A226V (Figure 5A, upper gel). Compared with the loading control gp64 protein (Figure 5A, lower gel), the expression of E1 from 26S-based constructs was lower than that from the monomeric-E1 constructs (Figure 5A vs. Figure 3A).

**Figure 6:**
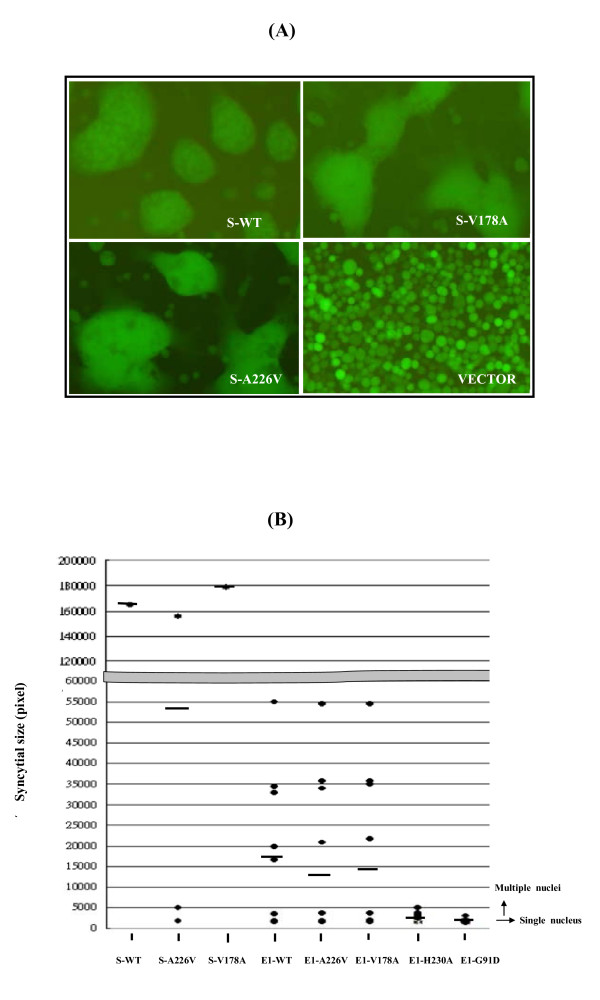
**Comparison of syncytial cell sizes**. **(A) **Sf21 cells were infected with indicated recombinant baculoviruses and cell fusions were induced as described above. Syncytial cell formation was examined under a fluorescence microscope with a green filter. The bar represents 50 μm. **(B) **Statistical summary of syncytial cell sizes induced by various recombinant baculoviruses as indicated. The vertical axis represents the sizes of fused cells and the horizontal axis indicates the various E1 constructs expressed by baculoviruses. The average size of syncytial cells induced by the indicated recombinant virus is indicated by a dash. The cell sizes of a single nucleus or multiple nuclei are indicated.

Immunofluorescence microscopy showed that all E1 proteins expressed from 26S-based constructs were present on the cell surface (Figure 5B) similar to results shown in Figure 3C. Examination of cell fusion ability under a fluorescence microscope showed that the average size of syncytial cells that expressed S-WT, S-A226V, and S-V178A was greater than that of those bearing E1 expressed by monomeric-E1-based constructs (Figure 6A vs. Figure 4A). We used the Image J software program to estimate the size differences between syncytial cells. The summarized data shown in Figure 6B clearly indicates that fusion cells induced by 26S-based constructs were 3 to 5-fold larger than those induced by monomeric-E1-based constructs were. A possible explanation for this is that E2 enhances E1 fusogenicity.

**Figure 7:**
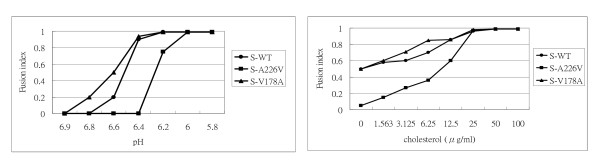
**pH and cholesterol-dependent profiles of cell fusion induced by CHIKV 26S-based E1**. **(A) **pH profiles. **(B) **cholesterol-dependent profiles. Methods are described in Figure 4B and 4C.

To determine whether structural proteins change the fusogenic capacity of mutated E1 protein with respect to pH and cholesterol dependence, we performed experiments similar to those described in Figure 4B. Our findings showed that the optimal pH for cells bearing S-A226V had shifted to pH 6 (Figure 7A), a lower pH than that for cells expressing E1-A226S (see Figure 4B). However, the cholesterol dependency of cells expressing 26S-based constructs required less cholesterol (25 μg/ml) (Figure 7B vs. Figure 4C). S-V178A consistently required less cholesterol than S-WT and S-A226V for cell fusion (Figure 7B). Thus, expression of other CHIKV structural proteins with E1 alters E1 fusogenic capacity.

## Discussion

In this study, we studied the roles of four amino acids of CHIKV E1 protein (G91, V178, A226, and H230) in cell fusion using an insect cell-based system [31]. This cell-based system revealed three important features of CHIKV E1 protein in membrane fusion. First, the highly conserved amino acid residues, G91 and H230, are important for membrane fusion functionality. Substitution of glycine with glutamic acid in the fusogen peptide disrupts the hydrophobic sequence, causing a loss of E1 fusogenicity (Figure 3A), which is consistent with the results of a study involving SFV G91D [27]. Although the histidine residue at E1 230 is located outside of the fusion sequence, the bending role of histidine cannot be substituted for by alanine. When we replaced the histidine residue, E1 fusogenicity almost vanished (Figure 4A).

Second, the less-conserved amino acid residues (V178 and A226) can be replaced without losing E1 fusion capacity (Figure 4A). However, substitution of these residues changes E1's fusion dependencies on pH and cholesterol (Figure 4B). Third, other CHIKV structural proteins influence E1 fusogenic capacity (Figure 6B). SFV *srf-5* mutants (V178A) are associated with decreased cholesterol-dependence on viral membrane fusion [22]. However, the CHIKV E1-V178A mutant exhibits changes in pH-dependence, but no significant differences in cholesterol-dependence occur. Our finding that CHIKV A226V mutant required more acidic pH conditions, together with greater cholesterol concentrations to trigger fusion activity (Figure 4C and Figure 7) could explain a previous report that infection with CHIKV A226V mutant is associated with a low viral titre compared to wild-type CHIKV infection of cholesterol-depleted C6/36 cells [18]. Limitations of an A226V mutant infection of cholesterol-depleted C6/36 cells may result in the viral fusion step having increased cholesterol-dependence. However, Tsetsarkin et al. demonstrated that CHIKV adaptation to *A. albopictus* mosquitoes does not correlate with acquisition of cholesterol dependence or low-pH thresholds for membrane fusion [38]. The observation of larger syncytia, induced by co-expression of E1 with E2, supports the observation that alphavirus E2 proteins both facilitate E1 folding and regulate E1 fusogenic properties, including cholesterol dependence.

Blissard and Wenz classified fusion induced by viral membrane fusion as "fusion from within" (FFWI), requiring viral fusogen synthesis. The authors defined "Fusion from without" (FFWO) as exogenous fusogen [39]. FFWI can be triggered by endogenous fusogen if delivered by authentic viral infection, transfection, or other virus-based vehicle. In this study, CHIKV E1 FFWI was triggered by a baculovirus-based vector containing a bicistronic co-expression system, so providing a new model that describes class II viral membrane fusion. The baculovirus-based vehicle is an efficient way to express fusogen on the cell surface without completion of the CHIKV replication cycle. A similar baculovirus-expression system has been used to express CHIKV E1 and E2 proteins in the development of a subunit vaccine to prevent CHIKV infection [40]. We applied the cell-based assay to compare the E1 fusogenicities of CHIKV and VEEV, and our findings were in agreement with a previous study that VEEV is insensitive to cholesterol depletion [41]. We also found that heparin and other polysaccharides could block cell fusion by VEEV E1 proteins, without inhibiting CHIKV E1-mediated membrane fusion (unpublished data). We therefore believe that cell-based assay using a baculovirus bicistronic vector is a safe and easy system for high throughput screening for agents that can block membrane fusion by CHIKV and other alphaviruses.

## Conclusions

In summary, we demonstrated that mutation of highly conserved amino acids of CHIKV E1 across 15 other alphaviruses, i. e., G91 and H230, lost the membrane fusion activity. In contrast, the less conserved amino acid residues (V178 and A226) were replaceable by other amino acid without losing fusion activity but changing in cholesterol and pH dependency slightly. The presence of other structural protein, E2, could enhance E1 fusion activity.

## Abbreviations

CHIKV: Chikungunya virus; SFV: Semiliki forest virus; SINV: Sindbis virus; VEEV: Venezuelan equine encephalitis virus; srf: Sterol requirement in function; MOI: Multiplicity of infection; EGFP: Enhanced green fluorescence protein; FCS: Fetal calf serum; dpi: Days post infection.

## Competing interests

The authors declare that they have no competing interests.

## Authors' contributions

SCK designed and performed the experiments and drafted the manuscript. YJC, YMW, PYT and MDK performed the experiments. TYW and SJL participated in the design of the study, data analysis, and writing the manuscript. All authors read and approved the final manuscript.
